# Anti-hyperglycaemic globulins from selected *Cucurbitaceae* seeds used as antidiabetic medicinal plants in Africa

**DOI:** 10.1186/1472-6882-13-63

**Published:** 2013-03-18

**Authors:** Clautilde Mofor Teugwa, Thaddée Boudjeko, Bruno Tugnoua Tchinda, Pascaline Chouadeu Mejiato, Denis Zofou

**Affiliations:** 1 Department of Biochemistry, Faculty of Science, Laboratory of Phytobiochemistry and Medicinal Plants Studies, University of Yaoundé I, P.O. Box: 812, Yaoundé, Cameroon; 2 Department of Biochemistry, Faculty of Science, Laboratoire de Phytoprotection et de valorisation des resources végétales, University of Yaoundé I, Biotechnology Centre, Yaounde, Cameroon; 3 Biotechnology Unit, University of Buea, Buea, South West Region, Cameroon

**Keywords:** *Cucurbitaceae*, *Telfairia occidentalis*, *Citrullus lanatus*, *Lagenaria siceraria*, *Cucumeropsis mannii*, *Cucurbita moschata*, Hypoglycaemic activity, Globulins

## Abstract

**Background:**

The recent epidemic of diabetes mellitus (DM) in Africa, coupled with rampant poverty, is an indication of the urgent need to develop new efficacious, cheaper and more available drugs to face this growing public health challenge. A number of plants products among which the protein-rich *Cucurbitaceae* seeds are commonly used in traditional medicine with increasing acclaimed efficacy against DM. The aim of this study was to analyse and evaluate the hypoglycaemic activity of storage proteins of five species of *Cucurbitaceae,* which include *Telfairia occidentalis, Citrullus lanatus, Lagenaria siceraria, Cucumeropsis mannii* and *Cucurbita moschata*.

**Methods:**

The different families of storage proteins were extracted following differential solubility, and their contents were estimated using the Bradford method. The analysis of these proteins was done by electrophoresis in non-denaturing and denaturing conditions. The evaluation of hypoglycaemic properties of various globulins extracted was performed on male *Wistar* rats by the oral glucose tolerance test.

**Results:**

The results showed that among the proteins extracted, globulins constitute the most abundant class of storage proteins in all five species selected. *Citrullus lanatus* and *Cucurbita moschata* presented the highest levels of globulin (275.34 and 295.11 mg/g dry matter, respectively). The results of electrophoresis showed that all species possess acidic and neutrals albumins and globulins, with molecular weight of protein subunits ranging from 6.36-44.11 kDa for albumins, 6.5-173.86 kDa for globulins and 6.5-49.66 kDa for glutelins. The 6.36 kDa of albumin subunit protein and the 6.5 kDa of globulin subunit protein were present in all the species. The oral glucose tolerance test showed that the globulins of the seeds of all species except *Cucumeropsis mannii* caused significant drop in blood sugar (88 – 137.80%, compared to the controls, p<0.05).

**Conclusions:**

These findings showed that the selected *Cucurbitaceae* seeds contained globulins with significant anti-hyperglycaemic activity. It is therefore highly encouraged to pursue investigations towards development of peptide-drugs and/or phytomedicines from these bioactive proteins which could be used as affordable alternative therapy against DM.

## Background

Diabetes mellitus (DM) is a syndrome of impaired carbohydrate, fat, and protein metabolism caused by either lack of insulin secretion or decreased sensitivity of the tissues to insulin [1]. The disease is characterized by hyperglycaemia, with fasting glucose greater than 1.26 g/L [2,3]. The American Diabetic Association (ADA) classified diabetes into four categories: type 1, type 2, gestational diabetes and diabetes associated with other specific conditions or syndromes [4]. In diabetic patients, failure to use glucose for energy ineluctably leads to increased utilization and decreased storage of proteins as well as fats, leading to metabolic acidosis [1]. Therefore, a person with severe untreated diabetes mellitus suffers rapid weight loss and asthenia despite the polyphagia. Without treatment, these metabolic abnormalities can cause severe wasting of the body tissues and death. Type 1 DM is due to beta-cell destruction leading to insulin deficiency. Therefore, insulin plays a key role in the etiology and control of type 1 DM [5]. Type 2 DM is caused by a combination of insulin resistance and relative insulin deficiency. It is thought that all forms of diabetes expose to early atherosclerotic heart disease and rapid aging. Diabetic patients are also at increased risk of ischemic heart disease, stroke, and peripheral vascular disease [1]. In most cases, the onset of type II diabetes occurs after age 30, often between the ages of 50 and 60 years, and the disease develops gradually. Therefore, this syndrome is often referred to as *adult-onset diabetes.* In recent years, however, there has been a steady increase in the number of younger individuals, some less than 20 years old, with type 2 diabetes. This trend appears to be related mainly to the increasing prevalence of obesity, the most important risk factor for type II diabetes in children as well as in adults. Together with hypertension and obesity, diabetes is among the top five continuing risk factors for cardiovascular deaths in the world [2]. DM is projected to exceed a prevalence of 380 million by the year 2030, with the type 2 accounting for about 90% of cases worldwide [6]. The disease is rapidly spreading in Africa today, as a result of rapid uncontrolled urbanization and westernization of lifestyle and dietary habits. Mbanya *et al.*[7] reported a prevalence varying widely across the continent: Benin 3%; Mauritania 6%; Cameroon 6.1%; Congo 7.1%; Zimbabwe 10.2%; Democratic Republic of Congo 14.5%. The situation in Africa, although not yet as worse as in most developed countries, is uniquely characterised by some alarming indicators. It is therefore a justified fear that DM with its accompanying generalized syndromes would become the next scourge in Africa if a particular attention fails to be taken both in prevention of the upset and the treatment of the disease. With this regards, like for many other diseases, the African herbal medicine represents a potential source of available source of new antidiabetic treatment easily affordable. Several plants have been shown to possess antidiabetic or hypoglycaemic properties, among which *Cucurbitaceae* family. In many African regions, *Cucurbitaceae* seeds are administered orally to treat diabetes and were previously reported to exhibit hypoglycaemic properties in mice [8,9]. *Cucurbitaceae* seeds are highly rich in proteins (*Telfairia occidentalis*: 33.2%, *Citrullus lanatus:* 30 – 35%, *Lagenaria siceraria*: 32.1 - 34.81%, *Cucumeropsis mannii*: 36.1 - 41.75%, and *Cucurbita moschata*: 29.33 - 35.88%) and globulin represents 60 – 90% of protein reserves [10,11]. The present study aimed at analysing the protein content of the selected five *Cucurbitaceae* species and assessing the ability of their globulin fractions to reverse induced hyperglycaemia in rats.

## Methods

**Figure 1:**
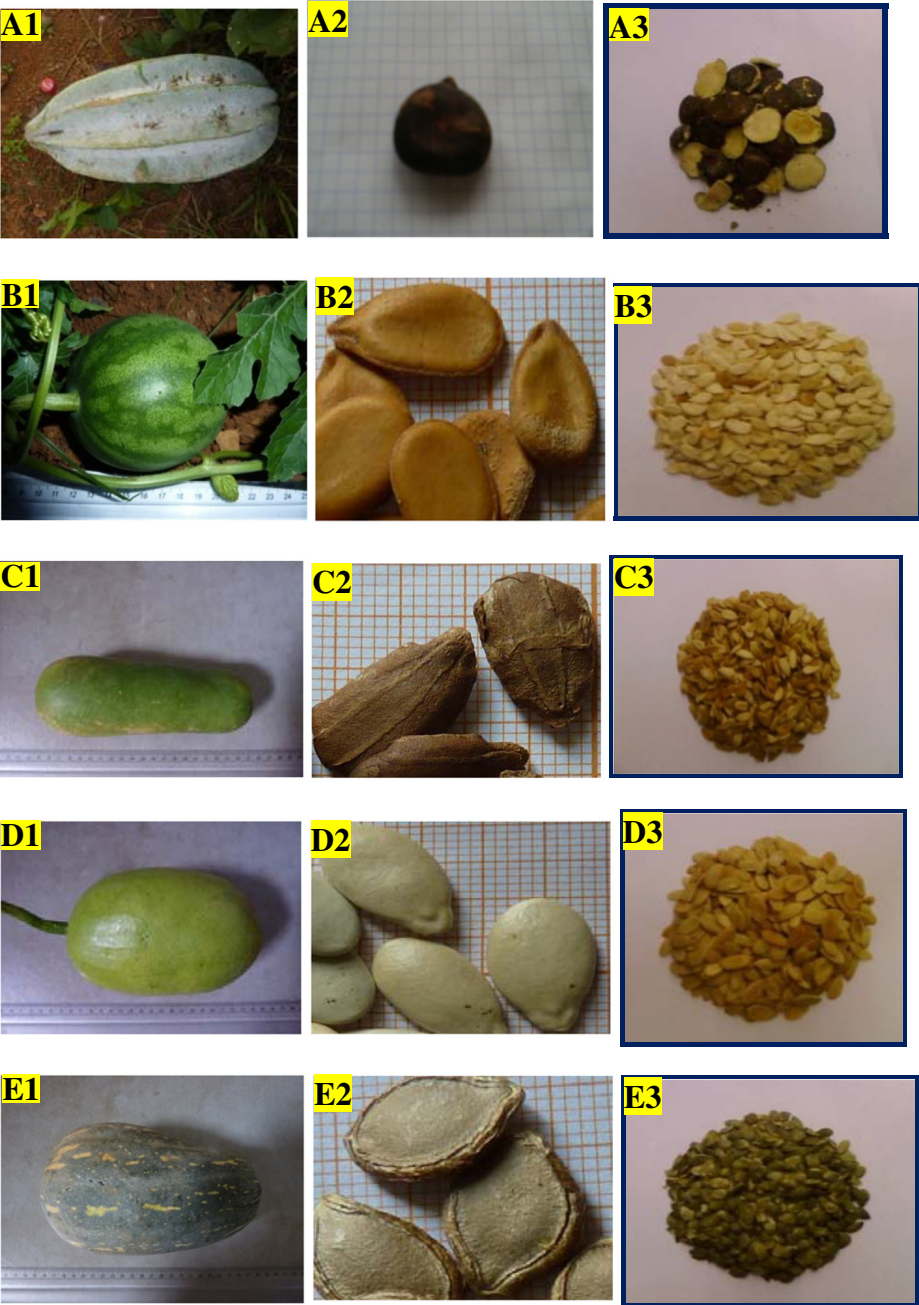
**Selected photographs of the seeds used in the study. A: **Photographs of *Telfairia occidentalis: ***A1. **Mature fruit, **A2. **Whole seed, **A3. **Kernel. **B:** Photographs of *Citrullus sp: ***B1. **Mature fruit, **B2. **Whole seed, **B3. **Kernel. **C: **Photographs of *Lagenaria siceraria: ***C1. **Mature fruit, **C2. **Whole seed, **C3. **Kernel. **D: **Photographs of *Cucumeropsis mannii: ***D1. **Mature fruit, **D2. **Whole seed, **D3. **Kernel. **E:** Photographs of *Cucurbita moschata: ***E1. **Mature fruit, **E2. **Whole seed, **E3. **Kernel.

The different seeds of *Telfairia occidentalis* (Voucher No: 33424/HNC), *Citrullus lanatus* (Voucher No: 42444/HNC)*, Lagenaria siceraria* (Voucher No: 8081/SRF-Cam)*, Cucumeropsis mannii* (Voucher No: 42485/HNC:) and *Cucurbita moschata* (Voucher No: 8106/SRF-Cam) were obtained from cultivated plants grown in an experimental garden on unfertilized soil set inside the University of Yaoundé Campus in the city town of Yaoundé, Cameroon (770 meters altitude). Figure 1 shows some photographs of the different fruits and seeds used in the present work. Upon collection, the identity of the plants was determined by the Cameroon National Herbarium in Yaounde, where voucher specimens were submitted and the identification numbers obtained (see above).

### Extraction and content analysis

The seeds were extracted from ripe fruits from different plant species, washed with water, and air-dried for about five days to facilitate kernels extraction. The dry seeds were then unshelled and the kernels dried at 50°C for two days. The dry kernels were subsequently ground to powder using an electric grinder (Moulinex, LM2221BM/890-0912R, China).

Prior to protein extraction, lipids were eliminated from the kernels powder by hexane, as previously described [12]. The residue obtained air dried at room temperature for 24 hr was weighed and proteins extracted from it as earlier described by Nasri and Triki [13]. Fifteen milligrams of residue was mixed with 1 mL distilled water at 4°C for 1 hr and then centrifuged at 10000 g for 20 min at the same temperature. The supernatant containing albumins was harvested, while the pellet was used in further extractions. It was rinsed with 0.5 mL distilled water before a 30 min homogenization, followed by centrifugation in the same conditions as in the previous step, to remove albumins completely. The pellet obtained underwent a similar series of steps (homogenization-centrifugation-rinsing) using a mixture of Tris HCl 100 mM in 0.5 M NaCl at pH 8.1 to extract globulins. The second pellet was submitted to a third and similar extraction of prolamins in 70%, and glutelins in acetic acid. The four protein groups obtained were quantified by Bradford method [14], lyophilized and stored at -20°C until required. They were then analysed using both non-denaturing electrophoretic techniques (BASIC-PAGE, 4%, pH 8.8) as previously described [15], and denaturing SDS-PAGE (12%, pH 8.8) according to the method of Laemmli [16]. The estimation of molecular weight was done based on the Pre-stained Protein Marker, Broad Range P7708S. At the end of the migration, gels obtained from the two types of electrophoresis were immerged for 2 hr, in a staining solution made up of methanol/acetic acid/distilled water (50/10/40, v/v/v) and Coomassie Brillant Blue R-250 at 0.25%. After staining, protein bands revealed were snapped using a numeric photo apparatus (Samsung), producing the electrophoregrammes.

### Hypoglycaemic activity testing (Oral Glucose Intolerance Test)

The hypoglycaemic activity of globulins from different *Cucurbitaceae* species was carried out in 3 month-old male *Wistar* albino rats with body weight of 285 – 310 g. The animals were obtained the Animal House of the Department of Animal Biology and Physiology, Faculty of Science at the University of Yaoundé I (Cameroon).

The lyophilized protein powders were used to assess the hypoglycaemic activity of the different globulins, following the method of Ariful *et al.*[17] with slight modifications. This method is based on the evaluation of the capacity of different globulins to decrease blood sugar following hyperglycaemia induced either by direct oral ingestion of glucose. At the start of the assay, 24 male rats were randomly divided into 6 groups of 4 each, and submitted to water and feeding at libitum for an acclimatization period of 7 days during which they were submitted to 12 hr light/dark cycle, and received water and food *ad libitum*. The 24 rats were thereafter submitted to a 12 hrs. fasting before and all through the period of experience. The glycaemia was determined on blood collected by incision of the tail end, using a Glucometer (Accucheck, USA). Rats of the control group (Group 1) received distilled water orally, while those of Groups 2, 3, 4, 5 & 6 received globulins solutions from *Telfairia occidentalis*, *Citrullus lanatus, Lagenaria siceraria*, *Cucumeropsis mannii* and *Cucurbita moschata* respectively. Each globulins solution was prepared by dissolving 80 mg of protein powder in 8 mL of distilled water. The proteins were administered *per os*, at 50 mg/Kg body weight. 30 min after globulin administration, a glucose solution (18 g in 45 mL) was administered orally in a single dose of 2 g/Kg body weight. Fasting blood sugar was determined at the beginning of the experiment, and subsequently, 30, 60, 90 and150 min after induction of hypoglycaemia.

### Statistical analyses

The data recorded both for the different proteins and experimental animals were expressed as Mean ± Standard Deviation and the different groups compared to each other using ANOVA and the Least Significant Difference (LSD) test. The analyses were conducted using Statgraphics Plus 5.0 at 95% confidence interval, and the graphical representations designed in Microsoft Excel 2007.

## Results

### Protein and lipid content of the different *Cucurbitaceae* species

Table 1 summarizes the proteins and lipids contents of the *Cucurbitaceae* species studied. Lipid content varies from 33.30% in *C. moschata* to 45.01% in *L. siceraria*. In general, globulins are the predominant protein class in all the five species, with *C. moschata* and *Citrullus lanatus* having the highest contents (295.11 and 275.34 mg/g defatted dried matter, respectively).

**Table 1:** **Protein and lipid contents of the different *Cucurbitaceae *species (mg/g defatted dried matter)**

**Content**	***Cucurbitaceae *species**				
	***Telfairia occidentalis***	***Citrullus lanatus***	***Lagenaria siceraria***	***Cucumeropsis mannii***	***Cucurbita moschata***
Albumin	28.00 ± 1.80^b^	40.48 ± 3.09^ab^	47.02 ± 20.28^a^	42.62 ± 2.57^ab^	32.64 ± 2.99^ab^
**Globulin**	**167.17 ± 3.63**^**b**^	**275.34 ± 28.31**^**a**^	**52.17 ± 5.68**^**c**^	**81.03 ± 3.93**^**c**^	**295.11 ± 38.94**^**a**^
Prolamin	9.92 ± 1.14^c^	18.21 ± 1.04^a^	11.767 ± 1.00^b^	8.63 ± 0.19^c^	8.58 ± 0.90^c^
Glutelin	41.67 ± 2.20^a^	6.83 ± 2.57^c^	4.53 ± 0.55^cd^	10.20 ± 1.93^b^	3.27 ± 0.61^d^
Lipids	37.78 ± 0.03^d^	39.49 ± 0.01^b^	45.01 ± 0.06^a^	37.94 ± 0.03^c^	33.30 ± 0.01^e^

**NB: **The letters a, b, c, d, e indicate whether there is any significant difference between species at 95% confidence interval. On the same line, values carrying the same letter are statistical similar.

### Electrophoretic analysis of the *Cucurbitaceae* globulins

#### Basic-page

**Figure 2:**
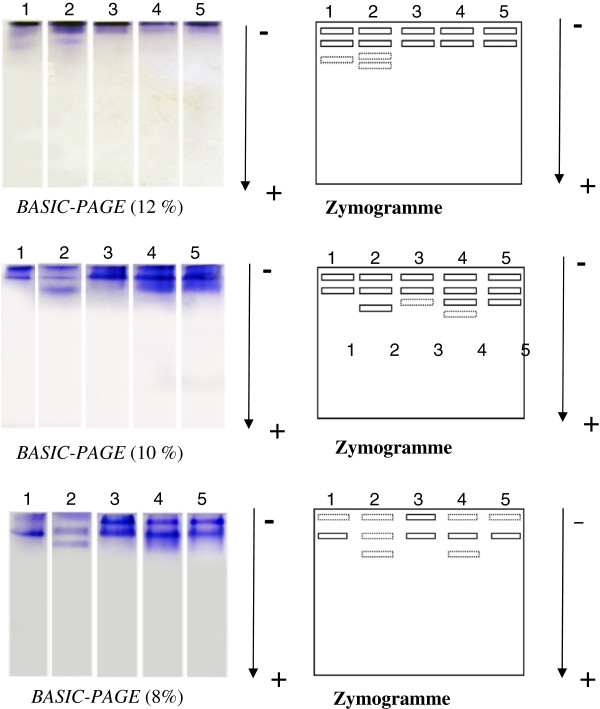
**Electrophoregrammes and zymmogramme of globulins from different *Cucurbitaceae *seeds in different concentrations of *BASIC-PAGE*. **1=*Telfairia occidentalis*; 2= *Citrullus lanatus; *3*= Lagenaria siceraria; *4*= Cucumeropsis mannii; *5*= Cucurbita moschata.*

The electrophoregrammes and zymmogramme of globulins from the selected *Cucurbitaceae* at different acrylamide concentrations are presented in Figure 2. It appears that all the seeds contained both acidic and neutral globulins. The ones from *C. mannii* and *Citrullus lanatus* are more diversified than those from *L. siceraria*, *T. occidentalis* and *C. moschata* which had only 3 bands each.

#### Sds-page

**Figure 3:**
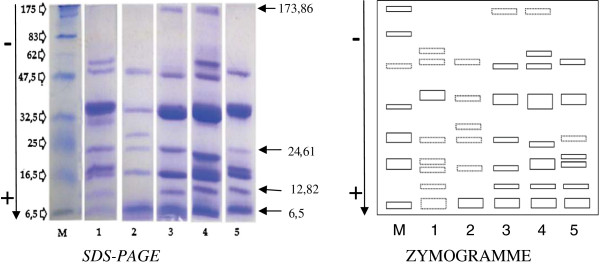
**Electrophoregrammes and zymmogramme of globulins from different *Cucurbitaceae *seeds in different concentrations of *SDS-PAGE*. **M = Molecular weight marker; 1=*Telfairia occidentalis *;2= *Citrullus lanatus; *3*= Lagenaria siceraria; *4*= Cucumeropsis mannii;* 5*= Cucurbita moschata.*

SDS-PAGE showed bands with molecular weight ranging from 6.5 to 173.86 kDa. The protein band at 6.5 kDa was present in all the five species. Those of 12.82 kDa and 24.61 kDa were also common, except in *Citrullus lanatus* and *C. mannii* (Figure 3). T. occidentalis had the highest diversity in globulin sub-types, with 9 bands (6.5; 12.82; 16.15; 17.49; 24.61; 30.58; 33.57; 56.93 and 60.57 kDa), followed by *C. mannii*, 8 bands at 6.5; 12.82; 16.95; 22.49; 32.16; 55.19; 60.57 and 173.86 kDa. The globulins from the seeds of *L. siceraria* and *C. moschata* appeared with 7 bands each. Their molecular weight were 6.5;12.82; 16.43; 24.61; 32.57; 55.19 and 173.86 kDa for *Lagenaria siceraria;* and 6.5; 12,82; 16,43; 17,49; 24,61; 32,57 and 56,93 kDa for *Cucurbita moschata.*

### Hypoglycaemic activity of the *Cucurbitaceae* globulins

**Figure 4:**
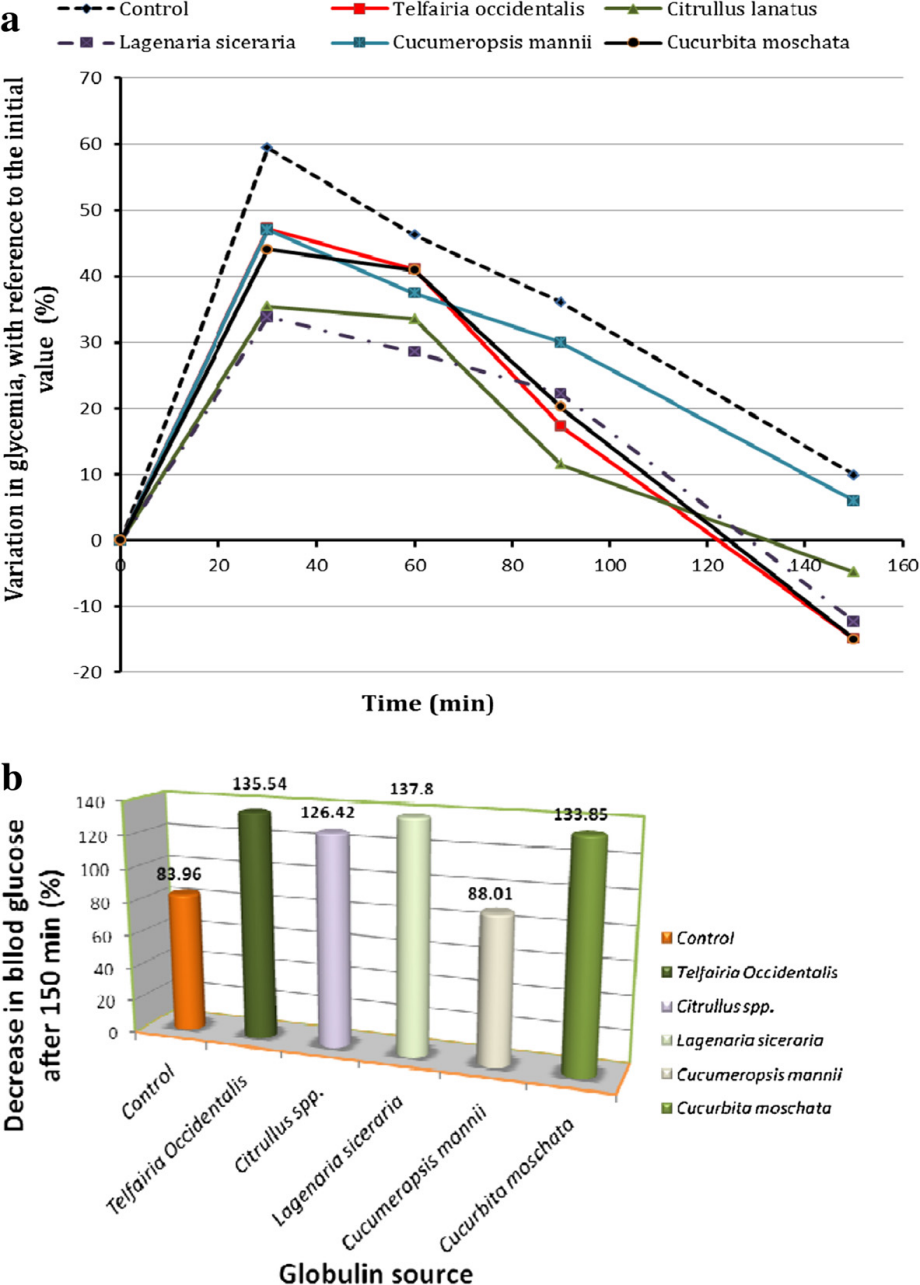
**Changes in glycaemia in different experimental groups during treatment. a:** Variation of glycaemia in different groups during the treatment. *The decrease was determined in % with reference to the initial value before glucose ingestion. ***b:** Decrease in glycaemia at 150 min, following hyperglycaemia induction in rats treated with globulins from different *Cucurbitaceae *species. *The decrease was determined in % with reference to the value obtained at 30 min following glucose ingestion.*

The induced hypoglycaemia, caused by glucose ingestion at the beginning of the experiment, decreased at different rates depending on the nature of the treatment administered to the rats. In general, the initial values were achieved 150 min post induction. With regard to the control group, the decrease became statistically more important in all the test groups after 150 min (p<0.05), except for *C. mannii* (Figure 4a). This decrease better illustrated by Figure 4b, clearly indicates values of 44.06; 40.96; 20.18 and -15.04% in *Telfairia occidentalis*, *Citrullus lanatus, Lagenaria siceraria* and *Cucurbita moschata* respectively, which are significantly more pronounced than those of the control group.

## Discussion

The aim of the present study was to determine the protein content of the selected five *Cucurbitaceae* species, analyse their globulin profiles, and assess their potential as source of antidiabetic peptide drugs.

The preponderance of globulins as main reserve proteins in the seeds of *Cucurbitaceae* was confirmed by the present study. The five species showed globulin content of 52.17 – 295.11 mg/g defatted matter. However, the value obtained for *C. lanatus* is slightly different from the 228.6 mg/g recorded by Ali *et al.*[18] working on cultivars from India. The differences noticed may be due to various possible factors like genetic and environmental changes. Likewise, the Cameroonian cultivars are particularly rich in term of globulin diversity, compared to the previous findings by Ali and co-workers, except for *Citrullus lanatus* where 6 bands were also observed. It is well documented that the nature of reserve protein is directly determined by the nature of enzyme isotype, the expression profile as well as the level of regulation of key enzymes involved in the synthesis and storage. While the nature of the enzyme isotype is intrinsic properties which can be alter by genetic modification, gene expression and regulation might be easily affected by environmental factors [19,20].

The seeds of *T. occidentalis* are commonly used in traditional medicine to treat a diversity of diseases including anaemia, convulsion, cardiovascular diseases and liver attack [21,22]. They are also administered after delivery to stimulate milk secretion in women [23]. The leaves were previously shown to possess hypoglycaemic properties [24-27]. The ethanol extract of leaves, seeds and whole fruits of *T. occidentalis* were shown to have hypoglycaemic activity [28]. From this study, it was observed that globulins from *T. occidentalis*, *C. lanatus, L. siceraria* and *C. moschata* significantly decreased fasting blood glucose in rates, following induced hyperglycaemia. Nmila *et al.*[29] showed that *Citrullus spp*. were rich in phenylalanine and leucine, and possessing insulin-stimulating properties. The globulins investigated in the present study may exert their activity using similar mechanism. Moreover, a peptide with significant biological activity (antifungal) was isolated from *T. occidentalis*[30]. Blouet [31] also observed that a mixture of essential amino acids could stimulate insulin secretion irrespective of gastrointestinal factors secreted during the digestion. This effect could be amplified by the digestibility of globulins, since van Loon *et al.*[32] and Calbet *et al.*[33] proved that insulin postprandial secretion in response to protein ingestion was influenced by the speed and the amplitude of the apparition of insulin-stimulating amino acids in the plasma. The juice from fruits of another Cucurbitaceae (*C. ficifolia*), was shown to significantly reduce fasting blood glucose in patients suffering from type 2 diabetes [34], thereby underlining the potential of *Cucurbitaceae* as anti-diabetics.

More interestingly, a sharp protein band at 24.61 kDa molecular weight was observed in the profiles of all four globulins profiles with significant anti-hyperglycaemic activity. The band was especially thicker in the species with higher activities, *T. occidentalis* and *L. siceraria*; and completely absent in *C. mannii* (less active). This particular protein which was present is likely to be the active peptide responsible for the activity observed. Further investigations are thus highly needed in order to confirm this hypothesis. The band should be extracted and test separately and the activity compared with both the ones of the other bands, and the whole globulin cocktail of the seeds.

## Conclusions

In conclusion, this study revealed the seeds of *Telfairia occidentalis*, *Citrullus lanatus, Lagenaria siceraria* and *Cucurbita moschata* as promising source of hypoglycaemic peptides, which deserve further investigations in order to validate and optimise the use of these species as antidiabetic medicines.

## Ethical considerations

Animals were handled according to the ethical guidelines of the Cameroon National Veterinary Laboratory (LANVET, Ministry of Livestock, Fisheries and Animal Industry) and the ARRIVE (Animal Research: Reporting *In Vivo* Experiments) guidelines.

## Competing interest

The authors declare that they have no competing interests.

## Authors’ contributions

CMT conceived the project and supervised the work all through, TB participated in protein purification and biological tests, BTT participated in plant collection, protein extraction and biological tests, PCM took part in biological tests, DZ participated in work design and drafted the manuscript. All the authors proofread and approved the manuscript before submission.

## Pre-publication history

The pre-publication history for this paper can be accessed here:

http://www.biomedcentral.com/1472-6882/13/63/prepub
